# Comparative Analysis of Edge Detection Operators Using a Threshold Estimation Approach on Medical Noisy Images with Different Complexities

**DOI:** 10.3390/s25010087

**Published:** 2024-12-27

**Authors:** Vladimir Maksimovic, Branimir Jaksic, Mirko Milosevic, Jelena Todorovic, Lazar Mosurovic

**Affiliations:** 1 Faculty of Technical Sciences, University of Pristina in Kosovska Mitrovica, Kneza Milosa 7, 38220 Kosovska Mitrovica, Serbia; vladimir.maksimovic@pr.ac.rs (V.M.); jelena.todorovic@pr.ac.rs (J.T.); 2 Academy of Technical and Art Applied Studies, School of Electrical and Computer Engineering, Vojvode Stepe 283, 11000 Belgrade, Serbia; mirko.milosevic@viser.edu.rs; 3 Directorate for Railways, Nemanjina 6, 11000 Belgrade, Serbia; lazar.mosurovic@gmail.com

**Keywords:** medical image analysis, feature extraction, edge detection, noisy images, object detection

## Abstract

The manuscript conducts a comparative analysis to assess the impact of noise on medical images using a proposed threshold value estimation approach. It applies an innovative method for edge detection on images of varying complexity, considering different noise types and concentrations of noise. Five edges are evaluated on images with low, medium, and high detail levels. This study focuses on medical images from three distinct datasets: retinal images, brain tumor segmentation, and lung segmentation from CT scans. The importance of noise analysis is heightened in medical imaging, as noise can significantly obscure the critical features and potentially lead to misdiagnoses. Images are categorized based on the complexity, providing a multidimensional view of noise’s effect on edge detection. The algorithm utilized the grid search (GS) method and random search with nine values (RS9). The results demonstrate the effectiveness of the proposed approach, especially when using the Canny operator, across diverse noise types and intensities. Laplace operators are most affected by noise, yet significant improvements are observed with the new approach, particularly when using the grid search method. The obtained results are compared with the most popular techniques for edge detection using deep learning like AlexNet, ResNet, VGGNet, MobileNetv2, and Inceptionv3. The paper presents the results via graphs and edge images, along with a detailed analysis of each operator’s performance with noisy images using the proposed approach.

## 1. Introduction

During the processing of an image, there is the tendency not to impair its quality and to generate as much information as possible. Sometimes, however, the quality itself is impaired at the exact source of images, too, i.e., at the moment when an image is being created, but not rarely so during its processing or transfer. Noises are frequent forms of this image distortion. Noises in an image represent unwanted information, and as such they cause consequences for the image, such as the occurrence of artifacts, a false edge and a false line, blurred objects, and the impairment of the image background itself as well. Noise is particularly undesirable in medical images for several reasons. Firstly, it reduces the diagnostic accuracy by obscuring the important details, making it difficult to identify conditions such as tumors. Secondly, it degrades the image quality, resulting in less sharp and clear visuals, which complicates the assessment of a patient’s condition. Thirdly, it requires additional effort from medical professionals to interpret the results, as they must distinguish useful information from the noise. The characteristic and model itself of noise can be represented via a histogram and the Probability Density Function (PDF) [1,2]. Different types of noise based on the PDF are Gaussian, Raileigh, uniform, impulse, Poisson, and so on. According to correlation, noise is classified as either white or colored. White noise has a balanced spectral density of the strength and zero correlation, differently from colored noise. If an image is impaired by white noise, it means that not all the pixels are interrelated. According to its nature, it is additive or multiplicative (speckle) noise, i.e., the pixels spanned by noise are either added to or multiplied by the reference image. According to the classification of sources, this is frequently called quantization noise or photon noise [3]. In the paper, the following noise types were used for the analysis of the performances of the new image edge detection approach, namely:

- Gaussian noise;
- Rician noise;
- Impulse noise (salt and pepper);
- Speckle noise.

### 1.1. Gaussian Noise

Because of its mathematical feature in the spatial and frequency domains, Gaussian noise models are often used in practice. Generally, Gaussian noise disrupts the level of the intensity of the gray color of pixels. For that reason, Gaussian noise is characteristic of its histogram or the PDF due to the dependence on the value of the gray color of pixels [1]. It is of a statistical and additive nature which follows a normal distribution with a zero mean value and the σ standard deviation and has an influence on all the image pixels. Its appearance is caused by the fluctuations in the temperature of the sensor and the variation in the illumination of the environment [3].

### 1.2. Impulse Noise (Salt and Pepper)

Impulse noise is additional noise most frequently appearing because of faulty sensors and an error during the transfer. Differently from Gaussian noise, it only affects certain pixels in the whole image, i.e., the image is not absolutely damaged, but only some pixels in it are. This noise type includes salt and pepper noise [1]. If, for example, a 3 × 3 matrix, with the pixels whose values range from 0 to 255 and there are 8 bits, is taken, if salt and pepper noise has hit the central pixel whose value was 250, now that value is close to zero, which means that it has become a dark pixel, whereas the rest of the pixels have remained unchanged. So, salt and pepper noise only affect certain pixels, and their values are replaced with dark pixels if that pixel was bright, i.e., if it was of a greater intensity and vice versa [1,3].

The intensity of this noise and its impact on the image quality are particularly significant in medical images, such as MRI (Magnetic Resonance Imaging) recordings, where even the smallest degradations can make diagnosis difficult. In the literature, there are methods that allow the objective measurement of the intensity of salt and pepper noise on MRI images. For example, the paper [4] introduces statistical analyses of local intensity extrema as a measure of image degradation. This approach provides the possibility to estimate the noise level without a reference image through the distribution of local maxima and minima, which are directly affected by the presence of impulse noise. Similarly, the paper [5] applies gradient methods to quantify the image quality, using variations in intensity as the indicators of noise and degradation. Although these methods are particularly effective for MRI images, the principle behind them can be applied to other types of medical images, including the CT and retinal images discussed in this paper.

### 1.3. Speckle Noise

This noise type is multiplicative noise. It frequently appears in coherent recording systems, such as the laser, radar, acoustics, and so on. Speckle noise in an image may appear in a fashion similar to Gaussian noise, yet it is far more difficult to observe by the observer since it makes it difficult to perceive the fine details in an image. Its probability density function follows the gamma distribution [2,5].

The noise in an image is usual and frequently present and is created at all the image levels, which can be seen based on the application of these three noise types as well. The most important part is the detection of edges over the images in which there is noise since the edge detectors such as Roberts, Sobel, and Prewitt, which are all based on the first derivative, are sensitive to noise [6,7]. For that reason, the Canny operator first filtrates the image, then performs detection. Many noise reduction filters are proposed, yet the filter type also depends on the noise type [8,9]. Numerous research studies are directed towards detecting edges in the images where there is noise, and many methods have been used to overcome this problem, recently most frequently using the artificial intelligence method and neural networks [3,7,10].

### 1.4. Edge Definition

The term “edge” implies a significant difference in the intensity of the gray color in the neighboring pixels. The image edge is where there is an abrupt change in the intensity between the neighboring or local pixels [8,9]. In this paper, an innovative approach to the assessment of the threshold value presented in [11] is analyzed. The approach was tested over the images consisting of a different number of details in the image where there is a different concentration (intensity) of noise. The detection of edges over the images in the presence of noise has become a big challenge. Therefore, edge detection in the presence of noise in the paper [12] was examined applying cellular neural networks (CNNs) and linear matrix inequality (LMI). The main work focuses on CNN training templates for noise reduction and edge detection [12]. Machine and deep learning are frequently used for the edge detection over the images with noise, as is presented in [13], where their model recognizes borders between the known and the unknown by pasting jittered negative patches over inlier training images. In the paper [14], an improvement of the Teaching Learning-Based Optimization (TLO) and a methodology for obtaining the edge maps of noisy real-life digital images are presented. Different image conditions can also be found in everyday image processing, so the adaptive detection approach to low-quality noise grayscale images is presented in the paper [15], whereas the paper [16] presents adaptive threshold selection in two stages for images with noisy low contrast. Researchers have also analyzed the influence of the changes in illumination and compression on edge detection using the Robert and Canny operators [17], while in the paper [18], an analysis of edge detection was performed using the Canny operator over noisy images. An analysis of the influence of different noise types on edge detection is presented in the paper [19], too, as well as the influence of multiplicative noise in various image types: synthetic aperture radar, ultrasound images, and ultrasonic imaging, among others [20]. In other words, deep learning methods, CNNs, have shown significant potential in edge detection and structure recognition in medical images. For example, the paper [21] presents an effective application of CNN architectures for detection optimization in industrial and biomedical applications. Although these methods are superior in terms of accuracy, their main drawback is the need for large datasets and computational power during training and inference, which limits their application in resource-constrained environments.

## 2. System Model

**Figure 1:**
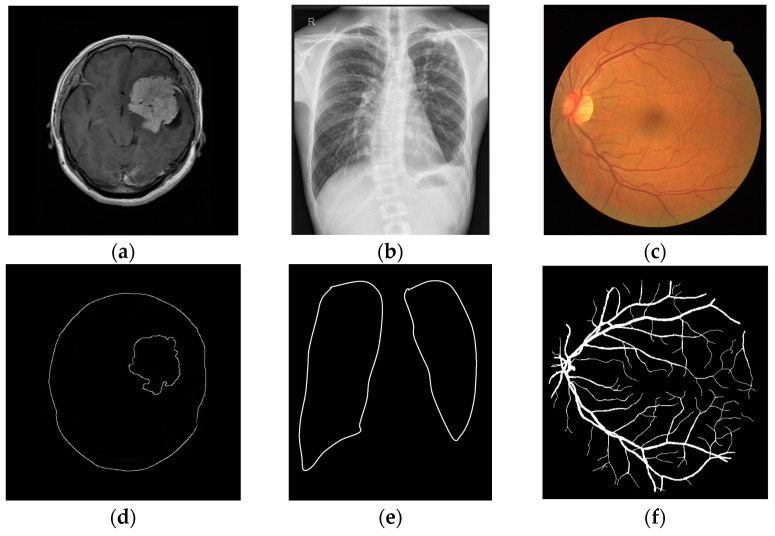
Example image for analysis: (**a**) small number, (**b**) medium number, and (**c**) large number of details, and ideal edges for (**d**) small number, (**e**) moderate number s, and (**f**) a large number of details.

Over 2000 images are analyzed in this paper, all of which were taken from medical databases (Retina, lung, and brain tumor segmentation) with their corresponding ground truth, and their complexity was assessed based on the mean value of spatial information (SI mean) [11]. Complexity is divided into three types of medical images and after that is confirmed via calculation by computing the spatial information values in each image applying the Sobel filter to the horizontal and vertical components of the image, and then calculating the standard mean value, standard deviation, and root mean square error [11]. Typically, SI mean is used as the primary measure because it has often shown the best results in predicting image complexity. Based on these values, three complexity criteria were established, namely low complexity (LD), medium complexity (MD), and high complexity (HD). In other words, boundaries were defined to represent a small number of details, a medium number of details, and a high number of details in the image. The newly proposed approach for estimating threshold values in [11] was tested in situations involving images affected by various types of noise and varying noise concentrations. Figure 1 illustrates an example of an image with low, medium, and high complexity, along with its ground truth.

In this manuscript, medical datasets of various modalities, including retinal images, brain tumor segmentations, and CT lung scans, were used to ensure the performance evaluation of algorithms on images with varying complexity, structure, and noise characteristics. Such a selection of datasets covers a wide range of details and enables the analysis of edge detection in real conditions of medical practice. In addition to these datasets, MRI datasets with specific acquisition parameters are also available, such as GRAPPA (Generalized Autocalibrating Partially Parallel Acquisition), which allow the variation in image quality depending on acquisition time [22]. In the paper [22], it was shown how different parameter settings can affect the textural features and noise level in MRI images. These data are significant for the evaluation of algorithms in the context of variable acquisition conditions, similar to how this paper analyzes the impact of different types and intensities of noise on the performance of edge detection in CT and other medical images. By using datasets with different modalities and image complexities, such as CT, retinal images, and MRI with quality variations, the specific challenges of edge detection can be further investigated, including the robustness of algorithms to noise and variability of the acquisition parameters.

Different reconstruction kernels in CT scanning, such as sharp and soft kernels, significantly affect the image characteristics, including the noise level. Sharp kernels emphasize edges and small details, but often increase the level of Gaussian noise, while soft kernels reduce noise at the expense of reducing the edge sharpness. This impact is described in the literature, which analyzes the transformation between sharp and soft kernels using filtering techniques. In this paper, we do not include an analysis of the effects of different kernels because our focus is not on the specifics of reconstructive algorithms in CT scanning, but on the generalization of the edge detection algorithm in regard to dense medical images.

**Figure 2:**
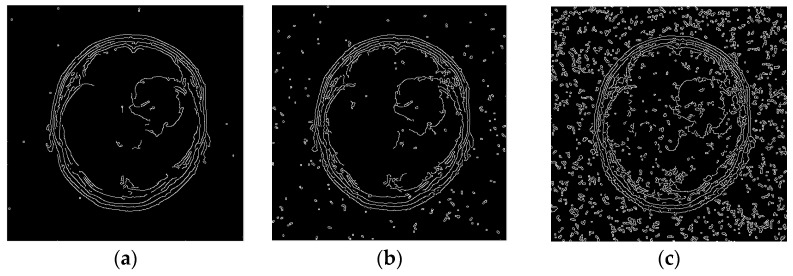
Example of edge detection on images affected by noise: salt and pepper with intensities of (**a**) 0.01, (**b**) 0.05, and (**c**) 0.1.

Edge detection serves to single out the desired objects in an image, for which reason as good detection as possible should be performed. A total of five edge detectors were used (Canny, LoG, Sobel, Prewitt, Roberts) over the images of different complexity and of different noise concentration (small, medium, and high noise intensity in the image). In the analysis, noise was added to each image, namely three noise types: salt and pepper, Gaussian, and speckle with the intensities of 0.01, 0.05, and 0.1. The objective measure (F–F1 Score) was used to verify the results [23]. FOM and PR objective measures were also computed, but for brevity of the manuscript, only the F measure is presented in the graphs. Figure 2 shows small (0.01), medium (0.05), and high (0.1) noise intensities for various types of noise and the Canny operator. The standard algorithm with default values for the Canny operator was utilized. It is evident that noise significantly affects edge detection, and further work will compare each edge detection operator for such images, with the addition of a new approach described in [11]. Additionally, all five operators will be applied using this approach.

In this analysis, the threshold assessment was determined using the machine learning technique, and the grid and random searches are presented in the paper [11]. A dataset of 1800 threshold values was created to achieve the best edge detection. There are 300 values for each single operator of the dataset, with a difference for the Canny operator that has two thresholds, so the Canny operator is assigned 600 values [11]. So, parameter optimization was achieved in two manners, namely via the grid and random searches [11,22].

The grid search (GS) is based on trying every combination of the parameters from the base, i.e., on going through all the dataset values and finding the best threshold value with the help of which the best edge detection will be achieved. The random search (RS) is based on setting the number of the parameters taken from the base randomly and testing the parameter that is the most appropriate one, based upon the objective quality detection assessment measures [11,23,24].

**Figure 3:**
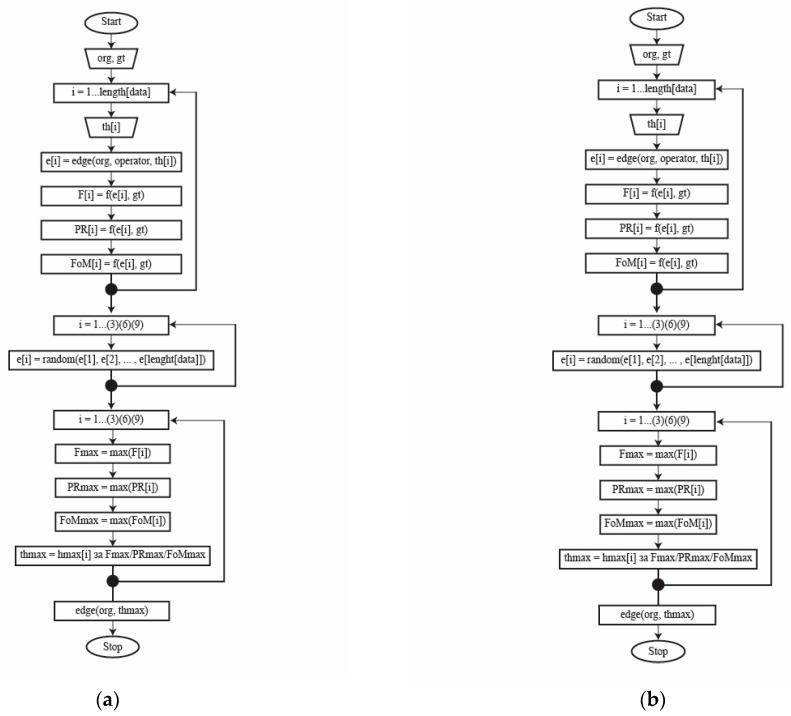
The flow chart for the proposed approach to threshold discovering based on (**a**) the grid search method, (**b**) the random search method.

The values 3, 6, and 9 were taken from the formed base for the random search, the best values of which are looked for that match the given threshold when detecting edges. So, while optimizing the parameters, the best model, i.e., the best parameter that will lead to the best edge detection is looked for. This parameter implies the determination of the threshold during detection, all based on the objective measures and the random and grid searches and the generated threshold values. The flowchart of the proposed approach in [11] is shown in Figure 3a for the grid search-based approach, and Figure 3b shows the random-based approach.

All the procedures for implementing the new approach were repeated and implemented in the same way and the same database for testing but now on the images affected by noise (different implementation situation). The flowchart of the approach in [11] is as follows:

Step 1: Loading the images from the dataset and ground truth images from the database. That is, the image org (image from the dataset) and the image with reference edges gt (ground truth image) are loaded.
Step 2: Loading the dataset with threshold values (th) of 100 values depending on the detector. For each detector, there is a dataset of 100 values. The Canny detector contains 200 values because it has two thresholds.
Step 3: Edge detection is performed over the images from the dataset (edge(orgI, th)), but th is selected using the GS method so that the dataset containing 100 values with thresholds is also selected for the best value, i.e., threshold th that gives the best edge detection. The threshold is selected by using the GS method by going through the entire dataset and taking the threshold that gives the best PR, F, and FoM value as the edge detection threshold. Objective measures require a reference image with an ideal edge (ground truth) and thus during the edge detection and finding the best threshold PR, F and FoM are obtained by comparing the ideal image with the detected edge and the image detected with the current threshold value.

When it comes to the RS3, RS6, and RS9 methods, unlike the GS method where all values from the dataset are searched to find the best value, here 3, 6, and 9 random values from the dataset are taken.

Step 4: The output is an image with the best detected edges.

**Figure 4:**
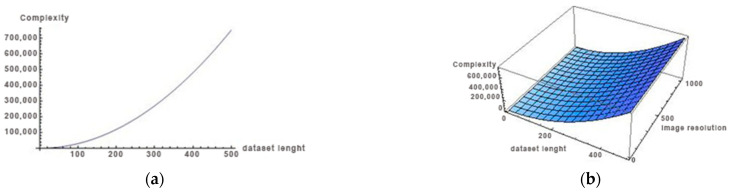
Algorithm complexity using GS and RS9: (**a**) 2D, (**b**) 3D.

Figure 4a,b show the algorithm’s complexity. As evidenced by Figure 4, this complexity hinges upon the level of detail within the image, specifically the resolution and dataset size. As the dataset values and resolution increase, the complexity escalates exponentially. The algorithm demonstrates robust runtime performance across the tested medical images dataset. Based on Figure 4, it is noteworthy that using the RS9 method yields notably high performance even with larger datasets. The findings of this study underscore the efficacy of RS9, recommending its application for optimizing algorithm performance with larger datasets.

The measured computational time for the GS and RS methods shows significant differences. GS requires an average of 2.15 s per image and with memory consumption of 10 MB, while RS reduces the time to 0.87 s with a minimal memory consumption of 0.01 MB. These results show that RS offers better computational efficiency for applications that require a fast response, while GS remains the more accurate choice for situations where time is not a critical factor. In terms of memory usage, GS and RS9 have minimal requirements because they work on 2D matrices, while deep learning models require significantly more resources due to the large number of parameters. Based on Figure 4, it can be concluded that larger datasets will significantly compare to the GS method; however, during the research, a larger dataset was also tested and it was concluded that the proposed method gives good results with the dataset used in the manuscript, which has 1800 thresholds. In other words, larger datasets achieve better results, but in real applications this is not necessary. Computationally, a time comparison between the traditional methods, the proposed GS and RS9 approaches, and the deep learning methods shows that GS is the most accurate but slowest, and RS is faster with controlled reduced accuracy. CNN models require more inference time on the GPU, but offer superior noise immunity and higher accuracy.

The proposed method of estimating thresholds using GS algorithms offers precise parameter setting for edge detection, but has certain limitations in terms of computational efficiency, while RS9 provides better optimization of the use of computer resources. GS, as a deterministic method, requires the examination of all the possible combinations of thresholds, which increases the execution time and memory consumption when working with high-resolution medical images or large datasets. In comparison, RS reduces the execution time with a trade-off in accuracy, as it randomly selects a subset of possible thresholds. The main challenge of the proposed method lies in its scalability to large datasets and high-resolution images. GS can become computationally inefficient, and future work envisages possible solutions that include parallel processing, the use of more efficient heuristic methods (e.g., Bayesian Optimization), or cloud computing.

## 3. Results

**Figure 5:**
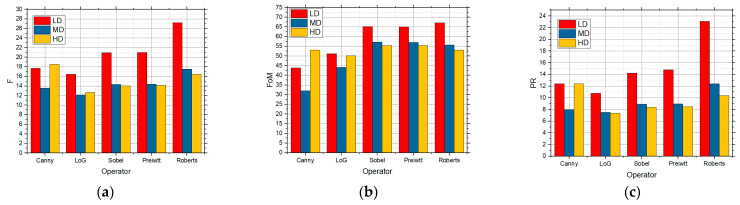
The values obtained by applying the standard approach for the images with LD, MD, and HD using the five edge detectors (**a**) F, (**b**) FoM, (**c**) PR values.

In Figure 5 [11], the results of edge detection at different image complexities are given. A total of five detectors (Canny, LoG, Sobel, Prewitt, Roberts) and three objective measures (F, FoM, PR) were used [9]. Based on the obtained values of these measures, the quality of the detected edge depends on the number of details. Based on that fact and the results accounted for in Figure 5, for LD images, the best edge detection was achieved by using the Roberts operator, but the Sobel and Prewitt operators generated similar results. As for the MD images, the Roberts operator led to the best results. The Canny operator was the best choice for HD images [11].

First, edge detection was performed using the standard algorithm, and then the proposed edge detection approach [11] was used, which selects the best threshold value based on the random and grid searches to perform the best possible edge detection.

**Figure 6:**
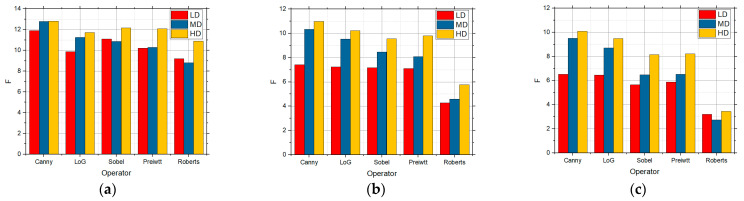
The F values obtained by applying the standard method for LD, MD, and HD images in the presence of the salt and pepper noise with the intensities of (**a**) 0.01, (**b**) 0.05, and (**c**) 0.1.

Figure 6 shows the F values for the LD, MD, and HD images over which edge detection was performed, and which contain in themselves the salt and pepper noise with the intensities of 0.01, 0.05, and 0.1, respectively. Detection was performed over these images for the five detection operators. According to Figure 6, the best was the Canny detector for all the three complexity levels. As the noise concentration increased to 0.05 (Figure 6b), Canny recorded the best results, although all those values were but slightly lower in comparison with 0.01 (Figure 6a), particularly so for the LD images. As the noise concentration increased to 0.1 (Figure 6c), the values were considerably lower, which means that the edge detection itself is worse as well. As in the previous cases, Canny recorded the best results, and noise considerably worsened the detection for the LD images. According to Figure 6, it can also be concluded that salt and pepper noise had an influence on the edge detection to a great extent, particularly so in the LD images.

**Figure 7:**
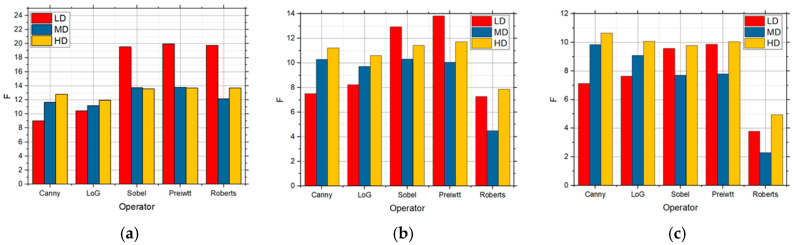
The F values obtained by applying the standard method for the LD, MD, and HD images in the presence of the speckle noise with the intensities of (**a**) 0.01, (**b**) 0.05, and (**c**) 0.1.

In Figure 7, the F values for the LD, MD, and HD images over which edge detection was performed and which contain the speckle noise with the intensities of 0.01 (Figure 7a), 0.05 (Figure 7b), and 0.1 (Figure 7c), respectively, are shown. In the case of the noise concentration being 0.01, the gradient operators recorded considerably better results for the LD images in relation to the LoG and Canny operators. These operators proved to be the better solution for both the MD and HD image as well. However, when there was a further increase in the level of noise in the image with the noise with the intensity of 0.05 and when the number of details in the image was small, Prewitt and Sobel recorded good results, whereas Roberts recorded considerably lower values, which can be seen in Figure 7. For MD and HD images, the Roberts operator recorded extremely bad results, particularly so for the MD images. For the HD images, all the operators, except for the Roberts operator, recorded quite similar results. Comparing it with Figure 5 which shows the absence of noise, it can be noticed that the results are satisfactory to a good extent. With high noise concentration and the speckle noise with the intensity of 0.1 is concerned, the Canny operator recorded the best results for the MD and HD images, whereas the Prewitt operator did so for the LD images. In this case as well, Roberts led to the worst results, i.e., the worst edge detection whose detection was not usable for further processing. In comparison with the lower noise concentration, the detection was the worst, i.e., lower F values were obtained, as expected.

**Figure 8:**
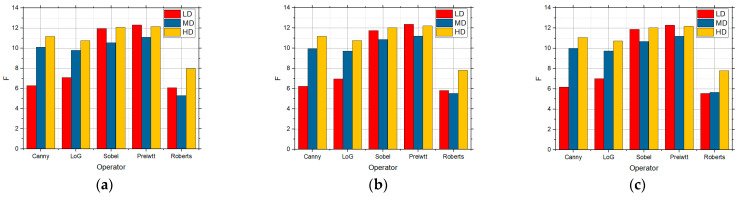
The F values obtained by applying the standard method for the LD, MD, and HD images in the presence of Gaussian noise with the intensities of (**a**) 0.01, (**b**) 0.05, and (**c**) 0.1.

Figure 8 shows the F values for the images with LD, MD, and HD over which edge detection was performed and which contain the Gaussian noise with the intensities of 0.01 (Figure 8a), 0.05 (Figure 8b), and 0.1 (Figure 8c), respectively. For noise with the intensity of 0.1, the best results were obtained by using the Sobel and Prewitt operators. For the MD images, the best results were obtained by using the Prewitt operator. The Roberts operator also recorded very bad results in this case as well. The increase in noise concentration to 0.5 and then to 0.1 (Figure 8b,c) allowed one to see the operator’s behaviors similar to one another to a great extent and the obtained edge detection values. The reason for this is attributed to the very model of Gaussian noise. Comparing it with Figure 5 showing the absence of any noise at all in the image, however, allows one to notice that Gaussian noise had a considerable influence on the edge detection for all the categories of complexity, but the most for the LD images.

If the noise types are compared with edge detection, it can be noticed that to a great extent, noise exerts an influence on the quality of edge detection. Salt and pepper and speckle influenced the LD images, particularly so when there was a greater intensity of noise. When salt and pepper noise is present, Canny proved to be the best operator for all the three complexity categories. Canny also generated the best results in the case of the speckle noise type for the LD and HD images, while the Prewitt operator provided the best results for LD images. When speaking about Gaussian noise, Prewitt was the best operator for all the three complexity categories.

### 3.1. The Results of Edge Detection by Applying the Proposed Approach Based on the GS Estimating Threshold Value Method

Unlike the previous cases, the proposed approach to the assessment of the value of the edge detection threshold was based on the grid threshold search method.

**Figure 9:**
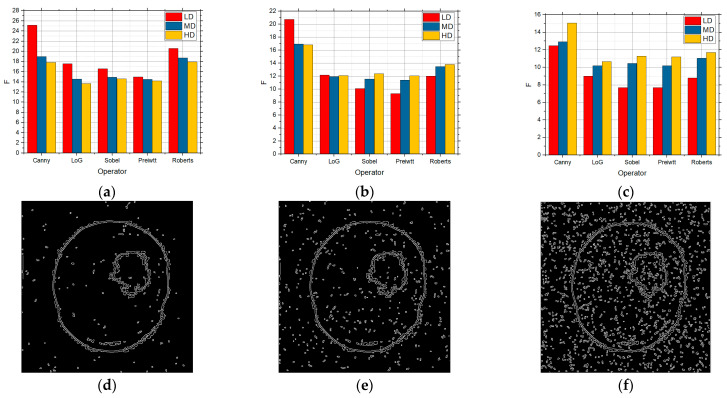
The F values obtained by applying the proposed approach based upon the GS threshold search method for LD, MD, and HD images in the presence of salt and pepper noise with the intensities of (**a**) 0.01, (**b**) 0.05, and (**c**) 0.1 and visual edge detection on that image using Canny operator for noise intensities of (**d**) 0.01, (**e**) 0.05, and (**f**) 0.1.

Figure 9 shows the F values for the LD, HD, and MD images over which edge detection was performed, and which contain the salt and pepper noise with the intensities of 0.01 (Figure 9a), 0.05 (Figure 9b), and 0.1 (Figure 9c), respectively. According to Figure 9, the best detection was achieved by applying the Canny operator for the LD images. For the MD and HD images, the best and similar results were obtained when applying the Canny and Roberts operators. By increasing the noise intensity in the image to 0.05, the results accounted for in Figure 9b were obtained. According to Figure 9, the best detection was achieved for all the three complexity categories by applying the Canny operator. There was also identical behavior noticeable for a high concentration of noise in the image, i.e., when there was the noise intensity of 0.1 in the image (Figure 9c).

However, when the noise intensity was 0.1 in the LD images, the values were lower in relation to the HD images, which was not the case when a lower concentration was present. Comparing these results with the results obtained when the algorithm without improvement and salt and pepper noise was used (Figure 6), it can be seen that a better edge detection was achieved to a great extent. Also, comparing this with the results shown in Figure 5 when the proposed approach was used but only over the images without noise, it can be seen that there were very good improvements, i.e., very good edge detection, even in the images with a high concentration of noise.

Due to the volume of the work, only the detection for the Canny operator is shown when the sum intensity was 0.01, 0.05, and 0.1. The images for the other edge detection operators are also available on request. Comparing this image with the results when the standard approach was used, it is noticed that when the intensity of the sum was small, and the number of details was medium, better results were obtained. As can be seen from the results, the best results were obtained for a small number of details in the image.

**Figure 10:**
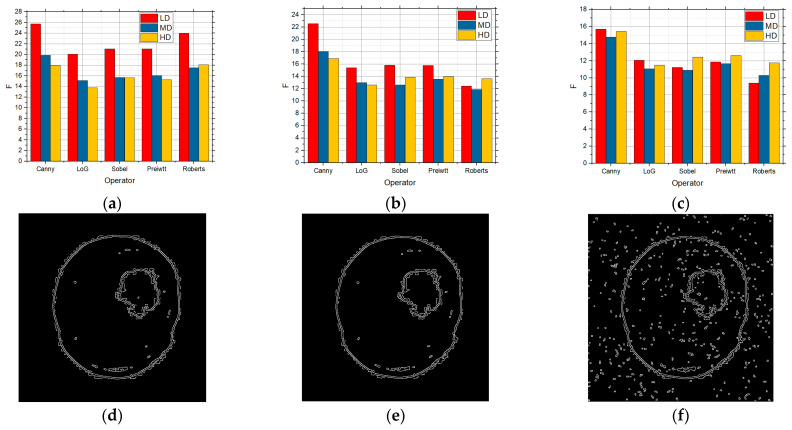
The F values obtained by applying the proposed approach based on the GS threshold search method for LD, MD, and HD images in the presence of the speckle noise with the intensities of (**a**) 0.01, (**b**) 0.05, and (**c**) 0.1 and visual edge detection on that image using Canny operator for noise intensities of (**d**) 0.01, (**e**) 0.05, and (**f**) 0.1.

Figure 10 shows the F values for the LD, MD, and HD images over which edge detection was performed, and which also had speckle noise with the intensities of 0.01 (Figure 10a), 0.05 (Figure 10b), and 0.1 (Figure 10c), respectively.

The best detection for all the levels of details in the image was achieved by applying the Canny operator for a low concentration of noise, i.e., 0.01 (Figure 10a). When the noise in the image had the intensities of 0.05 (Figure 10b) and 0.1 (Figure 10c), the best results were also recorded by using the Canny operator. Although the values were slightly lower in the case of a high noise concentration, better results were to a great extent obtained by applying the proposed approach based upon the grid threshold search method. If it is compared with the situation when there was salt and pepper noise for the LD images, the values were better in relation to speckle noise, so that salt and pepper affected the edge detection more, whereas the influence was to a great extent similar in the case of the MD and HD images. Figure 10d–f show the detection for the Canny operator for all three levels of speckle sum intensity in the image. Also, as in the previous case, the best results were achieved for a low intensity in the image, but were visibly better than the original approach.

**Figure 11:**
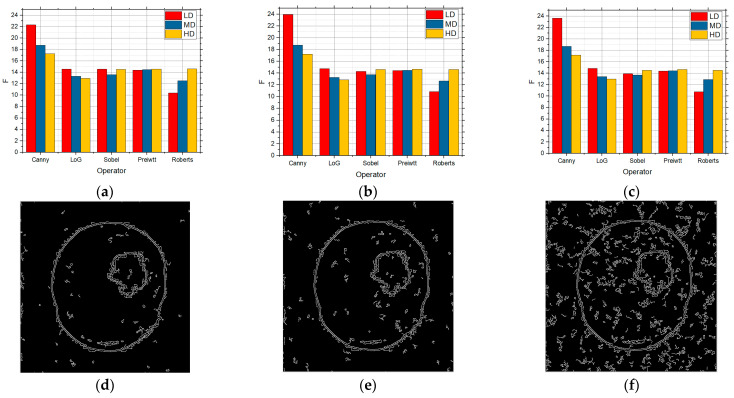
The F values obtained by applying the proposed approach based on the GS threshold search method for LD, MD, and HD images in the presence of Gaussian noise with the intensities of (**a**) 0.01, (**b**) 0.05, and (**c**) 0.1 0.1 and visual edge detection on that image using Canny operator for intensities of (**d**) 0.01, (**e**) 0.05, and (**f**) 0.1.

Figure 11 also shows the F values for the images with LD, MD, and HD over which edge detection was performed, which on their part also contain the Gaussian noise with the intensities of 0.01 (Figure 11a), 0.05 (Figure 11b), and 0.1 (Figure 11c), respectively.

As in the previous cases, the Canny operator recorded the best results for all the three complexity categories and for all the three noise intensity levels. In comparison with the previous noise types, a lower value was only recorded for the LD images, whereas better results were obtained for the MD and HD images when there was the Gaussian noise type with a great noise intensity in the image. The results show that even in this case, an improvement was made if detection is compared with the results when the proposed approach was not used. Comparing the results obtained for the Canny operator and when the Gaussian sum was present for all three intensities, the new method achieved significantly better results compared to the standard one, so it detected the edge very efficiently.

**Figure 12:**
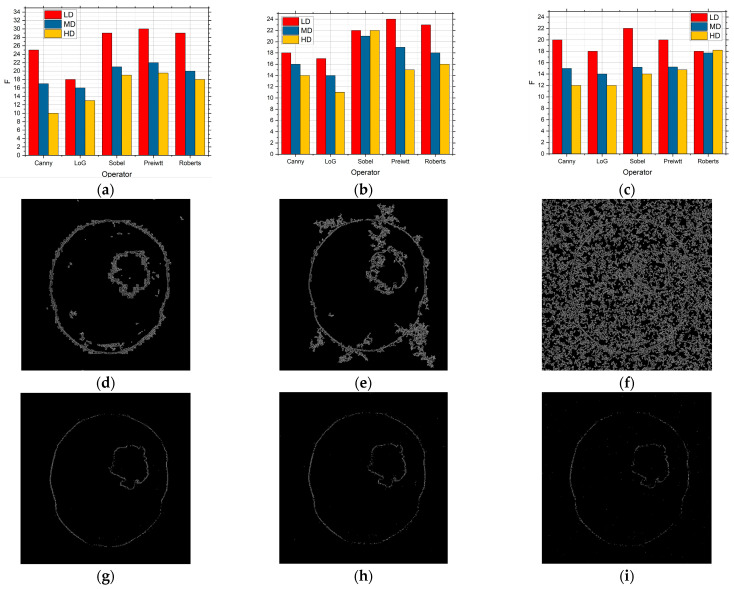
The F values obtained by applying the proposed approach based on the GS threshold search method for LD, MD, and HD images in the presence of Rician noise with the intensities of (**a**) 0.05, (**b**) 0.1, and (**c**) 0.15 and visual edge detection on that image using Canny operator for noise intensities of (**d**) 0.01, (**e**) 0.1, and (**f**) 0.15 and for Sobel (**g**) 0.05, (**h**) 0.1, and (**i**) 0.15.

Figure 12 shows what can be seen that when the Rician type of noise intensity was present. Figure 12 shows the results for (a) 0.05, (b) 0.1, and (c) 0.15, and the F values are shown. Figure 12 also shows the edge detection when the Canny operator was applied, while Figure 12g–i show the detection when the Sobel operator was applied for the described noise intensity. The results show that when Rician noise was present, algorithms based on a simple technique with masks such as Sobel, Prewitt, and Roberts gave better results than by using the Canny and LoG edge detection methods, especially when low- and medium-intensity noise was present. By comparing the one shown in Figure 12 with the results shown in Figure 9, Figure 10, and Figure 12, it can be seen that the GS method, when Rician noise of low and medium intensity is present, is much more effective than if methods such as Sobel, Prewitt, and Roberts are used. Also, the conclusion is reached when the GS method is effective and when a high intensity of Rician noise is present.

### 3.2. The Results of Edge Detection by Applying the Proposed Approach Based on the RS9 Estimating Threshold Value Method

This section presents the results of the testing of the proposed approach to the assessment of the values of the edge detection threshold based on the random threshold search method, i.e., on the nine random threshold values from the base. In the results obtained so far, this approach has proven to be quite efficient in respect to the execution speed and the obtained results.

**Figure 13:**
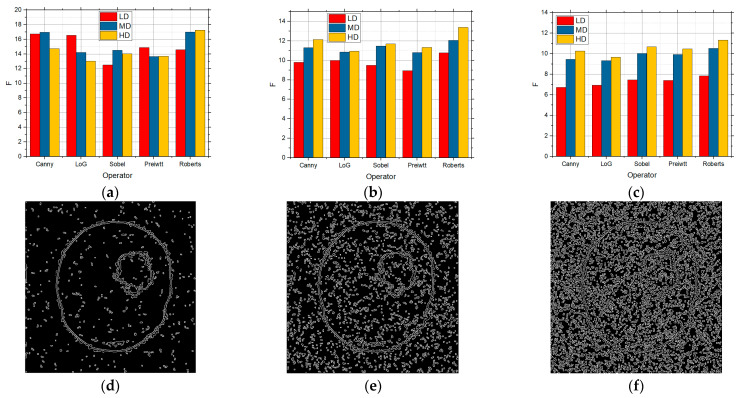
The F values obtained by applying the proposed approach based on the RS9 threshold search method for LD, MD, and HD images in the presence of salt and pepper noise with the intensities of (**a**) 0.01, (**b**) 0.05, and (**c**) 0.1 and visual edge detection on that image using Canny operator for noise intensities of (**d**) 0.01, (**e**) 0.05, and (**f**) 0.1.

Figure 13 shows the F values for the LD, MD, and HD images over which edge detection was performed, and which contain the salt and pepper noise with the intensities of 0.01 (Figure 13a), 0.05 (Figure 13b), and 0.1 (Figure 13c), respectively. Figure 13d–f show the detection for the Canny operator when it was the used approach based on nine random values for the threshold for the images with salt and pepper noise with 0.01, 0.05, and 0.1 intensities.

When there was a small 0.01 noise concentration in the image (Figure 13a), the best detection was achieved through the Canny operator for the LD and MD images, whereas the Roberts operator generated the best results for the HD images. Comparing it with the approach when three and six threshold values were used, the results were better when there were three values, but they were alike to quite an extent when six values were used. Further increasing the noise to 0.05 (Figure 13b) with this approach, the detection was better in relation to the three or six values for all the operators. The Roberts operator achieved the best detection for all the three complexity levels. Under the conditions of high noise with an intensity of 0.1 (Figure 13c), the results behaved in a comparable way to that for the detection of the six-threshold value approach. However, comparing the results presented in Figure 13, the values are greater by applying this approach. The best detector for all the three complexity categories is the Roberts operator, but the other operators also recorded comparable results. It should be mentioned that all these results are considerably better in relation to the situation when the standard approach was applied.

**Figure 14:**
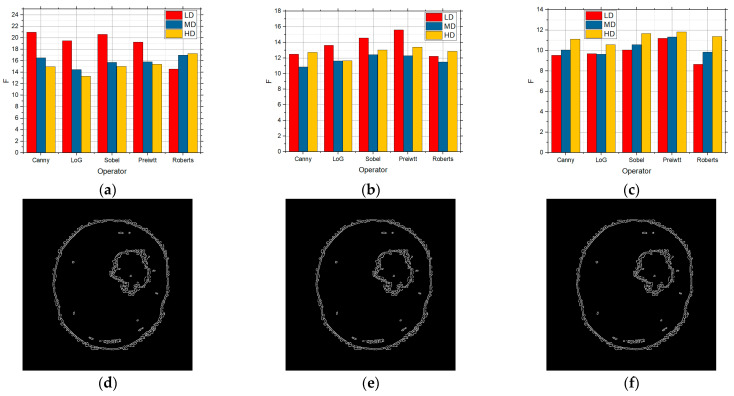
The F values obtained by applying the proposed approach based on the RS9 threshold search method for LD, MD, and HD images in the presence of speckle noise with the intensities of (**a**) 0.01, (**b**) 0.05, and (**c**) 0.1 and visual edge detection on that image using Canny operator for noise with intensities of (**d**) 0.01, (**e**) 0.05, and (**f**) 0.1.

Figure 14 also shows the F values for the LD, MD, and HD images over which edge detection was performed, and which also contained speckle noise with the intensities of 0.01 (Figure 14a), 0.05 (Figure 14b), and 0.1 (Figure 14c), respectively. Figure 14d–f show the detection for the Canny operator when it was the used approach based on nine random values for the threshold for the images with speckle noise with 0.01, 0.05, and 0.1 intensities.

The Canny detector recorded the best results when noise with the intensity of 0.01 of the speckle noise type in the LD images. However, good detection was also achieved by the other operators, except for the Roberts operator. The Roberts operator recorded the best detection for the MD and HD images. A further increase in noise to an intensity of 0.05 led to the detection in which the Prewitt operator achieved the best results for the LD images, whereas the Sobel, Prewitt, and Roberts operators recorded comparable results for the MD and HD images. Also, the Canny operator recorded good detection for the HD images. In the case when the intensity of noise in the image was 0.1, the Prewitt operator recorded the best detection for all the three complexity levels, but the values generated by the other operators were approximate for the HD images.

**Figure 15:**
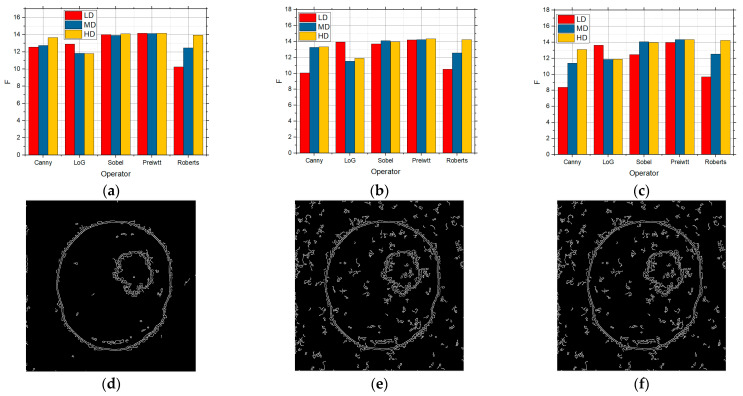
The F values obtained by applying the proposed approach based on the RS9 threshold search method for LD, MD, and HD images in the presence of Gaussian noise with the intensities of (**a**) 0.01, (**b**) 0.05, and (**c**) 0.1 and visual edge detection on that image using Canny operator for noise intensities of (**d**) 0.01, (**e**) 0.05, and (**f**) 0.1.

Figure 15 shows the F values for the LD, MD, and HD images over which edge detection was performed, and which were affected by Gaussian noise with the intensities of 0.01 (Figure 15a), 0.05 (Figure 15b), and 0.1 (Figure 15c), respectively. Figure 15d–f show the detection for the Canny operator when it was the used approach based on nine random values for threshold for the images with Gaussian noise with 0.01, 0.05, and 0.1 intensities.

When there was a noise intensity in the image of 0.01, Figure 15a allows one to notice that the values are to a great extent similar to when there were six random values, which can be seen in Figure 15a, but there is still improvement, particularly so in regard to the Canny operator. The Sobel and Prewitt operators achieved the best detection for all the three complexity levels. For the HD images, Canny and Roberts achieved almost equal detection. When the intensity of noise in the image was 0.05 (Figure 15b), the situation was quite similar even when there was a lower noise concentration (which was expected due to the very nature of noise) and exerted an influence on the Canny operator when there were LD images. When the intensity of noise in the image was 0.1 (Figure 15c), it could be noticed that it influenced the Canny operator and LD images the most, whereas the detection was similar for the other operators to what it was under the previous conditions of noise concentration for all the complexity categories.

**Figure 16:**
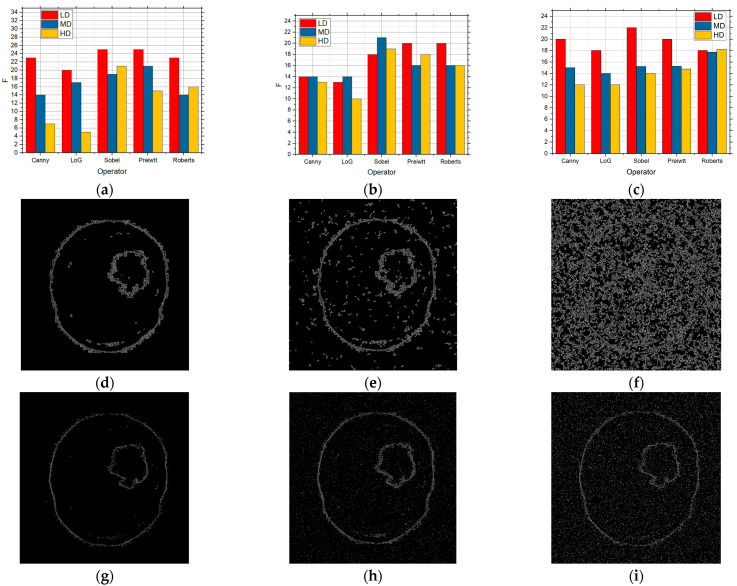
The F values obtained by applying the proposed approach based on the RS9 threshold search method for LD, MD, and HD images in the presence of Rician noise with the intensities of (**a**) 0.05, (**b**) 0.1, and (**c**) 0.15 and visual edge detection on that image using Canny operator for noise intensities of (**d**) 0.01, (**e**) 0.1, and (**f**) 0.15 and for Sobel (**g**) 0.05, (**h**) 0.1, and (**i**) 0.15.

Figure 16 shows what can be seen that when the Rician type of noise intensity was present. Figure 16 shows the results for (a) 0.05, (b) 0.1, and (c) 0.15 where the F values are shown. Figure 16 also shows the edge detection when the Canny operator was applied, while Figure 16g–i show the detection when the Sobel operator was applied for the described noise intensity. The results show that when Rician noise is present, algorithms based on mask techniques such as Sobel, Prewitt, and Roberts give better results than by using the Canny and LoG edge detection methods, especially when low- and medium-intensity noise was present. By comparing the obtained results with the results when the other types of noise were present, it can be seen that the proposed method using Sobel, Prewitt, and Roberts operators is more efficient than Canny and LoG.

Recent advances in edge detection algorithms emphasize deep learning techniques. Notable methods include DeepEdge, which combines classification and regression, and Holistic Edge Detection (HED) that employs multi-scale deep learning for hierarchical edge representations [25,26]. The solution named LPCB improves HED with VGG16 (Visual Geometry Group), while ECDN uses a convolutional codec for better edge localization. PiDinet introduces a pixel difference convolution operator, and CHRNet maintains high-resolution edge maps. Both papers show that Canny is the best option when the use of a regular algorithm is necessary, but the DL and ML approaches give better results [27]. In [28,29], more focus was gained to overview and review the state of the art in edge detections using regular and DL algorithms. The paper [30] used Dynamic Threshold Neural P Systems with Orientation (ODTNP), a neural-like computing model designed to overcome the common edge detection shortcomings such as discontinuous edges, weak edges, noise sensitivity, and difficulty in setting the gradient thresholds. In [25,26,27,28,29,30], the BSD500 dataset and performance metrics such as precision, recall, F-score, ODS, and OIS are used for evaluating these algorithms’ effectiveness in edge detection, but there is no computational time for these algorithms and approaches. In [7], a method is proposed to address the challenge of edge detection in noisy digital images. The approach involves partitioning the image into equal segments and calculating an initial threshold for each segment based on the highest frequency of brightness intensity rather than the mean brightness, which is adversely affected by noise. The experimental results demonstrate the method’s effectiveness, with a PSNR (Peak Signal to Noise Ratio) of 61.4896 for one test image compared to lower values achieved by the baseline methods. This indicates the proposed method’s robustness in maintaining the edge continuity and clarity despite the presence of noise, but there are only PSNR metrics which are not fully reliable. Also, there is no computational time. As Canny proved to be the best edge operator in [31], the authors reconfigured the Canny detector and its hardware realization for noisy images.

**Figure 17:**
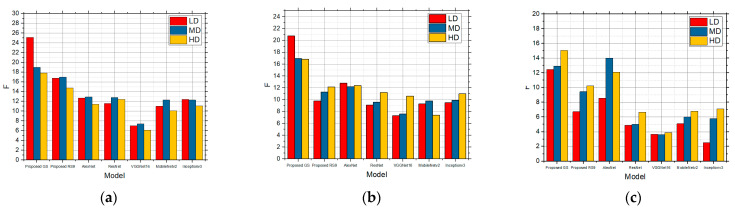
Comparison of proposed approach and other approaches using Canny edge detection on the noisy image affected by salt and pepper: (**a**) low intensity, (**b**) medium intensity, and (**c**) high intensity.

**Figure 18:**
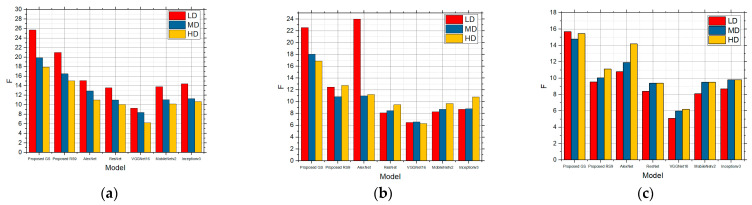
Comparison of proposed approach and other approaches using Canny edge detection on the noisy image affected by speckle: (**a**) low intensity, (**b**) medium intensity, and (**c**) high intensity.

**Figure 19:**
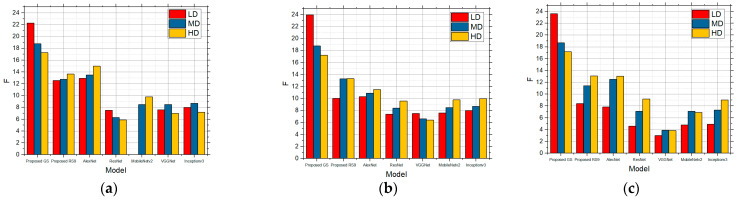
Comparison of proposed approach and other approaches using Canny edge detection on the noisy image affected by Gaussian: (**a**) low intensity, (**b**) medium intensity, and (**c**) high intensity.

Although the grid and random search approach optimizes the threshold values efficiently, the computational cost can still be significant, especially for high-resolution images or large datasets, especially if using the GS method. Based on the results where the images from the medical dataset were used and where different sum intensities were used, a large number of examples of images found in everyday cases were covered. Datasets have images that are from real examples, so by categorizing them into groups by the different numbers of details and different intensities of noise, this algorithm becomes meaningful. However, using a larger number of images leads to better results, especially using the GS method, but this is also a potential limitation of the algorithm because it may require considerable memory resources to store and process the numerous threshold values and intermediate results, which can be a constraint on systems with limited memory capacity. The proposed method has been primarily tested on the BSD dataset and after that on the medical datasets, which consist of real example images with specific characteristics. Its performance on other types of images, such as medical images, satellite images, or images from other domains with different noise characteristics, remains to be studied in future directions, especially when noise and compression are present. Considering that a larger database of images, especially specialized images, can be tested, the future direction may be the specialized optimization of the algorithm under conditions of images when noise or compression is present. Therefore, the optimization and application of parallel processing techniques can help distribute the computing load across multiple processors, reducing the time required for optimization. This may involve using GPU or cloud-based computing resources. For each image category, the edge detection performance of five models was evaluated: AlexNet, ResNet, VGGNet, MobileNetv2, and Inceptionv3. Performance was measured using the F-score metric, where images were exposed to different types of noise and different intensities. Figure 17 shows a comparison of the salt and pepper noise intensities of (a) low (0.01), (b) medium (0.05), and (c) high (0.1). Figure 18 shows a comparison for speckle noise intensities of a) small (0.01), (b) medium (0.05), and (c) large (0.1). Figure 19 shows a comparison of Gaussian noise intensities of (a) small (0.01), (b) medium (0.05), and (c) large (0.1). Each of these models has specific architectural features that affect their edge detection ability and noise immunity. AlexNet is one of the first deep convolutional neural network (CNN) models to achieve significant success in the ImageNet competition. AlexNet has a relatively simple architecture with eight layers (five convolutional and three fully connected layers). This model is good for basic detection tasks, but may have limitations in more complex scenarios. ResNet is known for its 50-layer deep architecture, using “residual” connections that allow gradients to flow more efficiently through the network. This model is very robust and efficient in complex detection and classification tasks. VGG-16 is a deep convolutional network with 16 layers, known for its simple and uniform architecture. All the convolutional layers use 3 × 3 filters, which allows for the more accurate detection of the local features. However, this model may be sensitive to noise due to its deep structure. MobileNetv2 is an optimized version of the MobileNet architecture, which uses “depthwise separable convolutions” to reduce the number of parameters and computational requirements. This model is ideal for applications on resource-constrained devices, but may show different performance depending on the type of noise. Inceptionv3 is a complex model that uses multiple convolutional filters of different sizes in each layer. This architecture allows for better edge detection in the presence of complex noises, but can be computationally demanding.

The proposed approach shows a significant advantage in edge detection compared to other methods. The difference in the quality of edge detection between the proposed approach and alternative methods is visible. The proposed model retains more details and has less noise, which shows better efficiency. Although the proposed approach shows a better edge detection ability in most cases, the high intensity of salt and pepper noise presents a significant challenge. In some cases, alternatives such as methodologies based on median filtering or adaptive thresholding may show better robustness to the high intensity of this type of noise. Such approaches can preserve more detail in some scenarios, especially when the noise intensity is extremely high. Although the proposed model shows solid performance, at a moderate Gaussian noise intensity, methods that combine Gaussian filtering with sophisticated edge detection algorithms can show better results in certain aspects, such as edge smoothness and noise reduction without a significant loss of detail. As the noise intensity increases, the performance of all the methods decreases. However, the proposed approach still shows good results. The difference in performance is still significant, but slightly less pronounced than at a low intensity. At a high noise intensity, all the models experience a large drop in performance. Nevertheless, the proposed approach manages to maintain relatively better results, with clearer edges and less noise. The difference compared to other models is smaller, but still significant. In these cases, methods using advanced speckle noise filtering techniques, such as Wavelet decomposition, can provide better results in certain aspects, such as preserving the edge structure while reducing noise.

There are more advanced models within machine learning that belong to the subcategory of deep learning such as DexiNet (Dense Extreme Inception Network) [32], LDC (Lightweight Dense CNN) [33], or CATS (Context-Aware Tracing Strategy) [34]. DexiNet is a deep learning model designed for edge extraction in images, known for its extremely detailed edge detection. It uses an architecture inspired by Inception blocks, but is modified to include dense connectivity and multiscale feature extraction. The goal of the model is to identify the edges of objects in images with a higher level of detail, especially in scenarios where the edges are thin and indistinct. They are used for multiscale analysis, allowing the model to identify edges at different resolutions. Each layer is connected to all the previous layers, thus improving the feature propagation and optimization during training. Thanks to the densely connected architecture and multiscale analysis, it is suitable for images with a large number of details, but also for images covered by forest. However, due to its architecture, DexiNet requires significant processing resources [32]. Even better results are shown by the LDC method, which is also based on the DexiNet algorithm; however, in order to seek a better compromise between performance and application, a smaller filter size and compact modules were considered. As a result of the modification, a model with less than 1 M parameters is obtained, which is fifty times smaller than DexiNet, as well as lighter than most state-of-the-art approaches [33]. CATS is a model based on a context-aware tracking strategy for ivic detection based on an observation that the localization ambiguity of deep edge detectors is mainly caused by the mixing phenomenon of convolutional neural networks: feature mixing in edge classification and side mixing during fusing side predictions [34]. The results in the paper [34] show that CATS provides better detection than RCF (Richer Convolutional Features) and BDCN (Bi-Directional Cascade Network) by factors of 12% and 6%, respectively, when evaluating without using the morphological non-maximal suppression scheme for edge detection [34]. AI models have demonstrated state-of-the-art performance in edge detection tasks, especially in complex and noisy images. However, it is important to note that AI-based solutions, particularly deep learning models, are not entirely robust or safe. Recent studies have demonstrated that even minor perturbations, such as changing the value of a single pixel, can drastically alter the results of classification or edge detection. This phenomenon, known as adversarial attack, raises concerns about the reliability of AI in sensitive domains like medical imaging. For instance, in [35], it is highlighted how adversarial attacks could compromise medical image classification, emphasizing the need for robust AI models that are resilient to such manipulations. This represents an important challenge for future work, particularly in ensuring the robustness of AI-based edge detection methods under adversarial conditions.

DL models like DexiNet, LDC, and CATS and hyperparameter optimization methods like Random Search and Grid Search discussed in the paper cannot be directly compared because they have fundamentally different purposes and functions within machine learning. DL models are complex architectures designed for specific tasks such as edge detection, semantic segmentation, and spatio-temporal analysis, where the model is trained on large amounts of data to directly achieve specific results. On the other hand, Random Search and Grid Search are techniques used to optimize the hyperparameters within a model, regardless of which model is used, with the aim of improving the performance of already existing algorithms. While DL models learn from data and directly affect the performance in specific tasks, Random Search and Grid Search only optimize the model parameters and are not directly related to the learning and data processing process, but indirectly. Table 1 shows the characteristics of the model for parameter optimization and the model that directly affects edge detection.

Edge-preserving filtering such as Sigma Filter, Anisotropic Diffusion, Bilateral Filter, and Non-Local Means (NLMs) are widely applied in noise reduction while preserving the key desired information. Although these filters effectively reduce the noise in images, their application can significantly affect the optimization process of the edge detection algorithm. Including additional filtering before edge detection introduces an additional step that can upset the existing balance between the computational complexity and detection accuracy. Considering that the Canny algorithm showed the best results while preserving performance, it is recognized as a proctor for improving the existing preprocessing such as regarding the difference between an original image and the image processed using edge-preserving filtering. The focus of this work was on the direct impact of noise on the performance of edge detection algorithms, thus avoiding additional interventions that could obscure the interpretation of the results and the effectiveness of the proposed approach. Nevertheless, the difference between the original image and the image filtered by using edge-preserving techniques can provide additional information which is useful for improving edge detection, especially in environments with a high level of noise, and the absence of such filters can be cited as a shortcoming of the algorithm. The integration of these techniques with the proposed method represents a promising direction for future research, where their influence on the trade-off between noise reduction, edge preservation, and computational efficiency of the algorithm would be examined in detail.

## 4. Contributions

The results make a contribution to the field of medical image processing, with a special emphasis on edge detection in images affected by different types of noise. The key contributions of the paper can be summarized as follows:

Two proposed methods were tested for evaluating the optimal edge detection thresholds over medical images affected by different types and intensities of noises that are mainly found in nature images.

First, a GS method that searches the threshold parameter and provides the most accurate edge detection results was tested. The testing has shown that GS achieves the maximum F-measure even for highly complex images (with a high number of details), which confirms its accuracy in difficult conditions.

Second, the RS9 method significantly reduces the processing time (0.75 s per image) with minimal memory usage (0.01 MB), providing a balance between efficiency and accuracy. This contribution is particularly significant for real-time applications and applications in systems with limited computing resources.

Also, a comparative analysis of the performance of five traditional detectors (Canny, LoG, Sobel, Prewitt, and Roberts) on medical images affected by different noises is presented: salt and pepper, speckle, Gaussian, and Rician noise. The Canny operator achieves the best edge detection accuracy on images with Gaussian and speckle noise, especially when a high complexity exists. The Sobel and Prewitt operators show greater resistance to Rician noise, while the results are stable on images of medium complexity. The Roberts operator gives the most efficient results for low-complexity images, with a significantly shorter execution time. These results provide a clear framework for algorithm selection depending on the complexity of the image and the type of noise present.

Compared with deep learning methods, the proposed approach shows the following results: With regard to the execution time, GS requires 7.35 s per image, while RS9 achieves processing in 0.75 s, thus confirming its suitability for applications that require fast data processing. For memory load, the RS9 has negligible memory usage (0.01 MB), while DL models require significantly more resources due to the large number of parameters and the need for GPU inference. By comparison with CNN models (e.g., U-Net, DexiNet, and LDC), it was concluded that the traditional threshold optimization methods, especially RS9, offer a sufficiently high accuracy with far lower computational usages. By testing on images with three levels of complexity (low, medium, and high) and different noise intensities (0.01, 0.05, and 0.1), the robustness of the proposed methods was confirmed. Threshold optimization enables efficient edge detection even in high-noise images, thus contributing to the better segmentation of structures in medical images. The research results are directly applicable in medical image processing.

The proposed approach can improve edge detection, especially when there is noise in the image, for example the detection of blood vessels in retinal images, which is crucial for the early recognition of diabetic retinopathy or the segmentation of tumor edges in brain MRI images, enabling the more precise monitoring of changes or the detection of nodules in lung CT scans, which facilitates the early detection of malignancy. The proposed approach shows robust performance even in the presence of noise, while its efficiency in terms of time and memory requirements opens possibilities for application in systems with limited resources, as well as in real-time applications for medical diagnostics.

As part of the contribution, the potential directions for further development are identified, such as the integration of an edge-preserving filter (Bilateral Filter, Anisotropic Diffusion) to improve the edge detection in images with extreme noise levels. The optimization of the execution time can be achieved through the parallel processing and application of Bayesian Optimization algorithms. The development of hybrid systems that combine the advantages of traditional algorithms with deep learning methods can achieve an optimal balance between the accuracy and speed. One of the potential limitations is the longer execution time of the GS method with larger datasets. Nevertheless, the research results show that the proposed number of thresholds and the tested dataset provide good enough results, and it is not necessary to additionally increase the volume of datasets for practical applications (except in cases where a specific application requires it). On the other hand, the RS9 method shows significantly less sensitivity to the size of datasets and successfully maintains the efficiency, even when applied to larger datasets.

## 5. Conclusions

In the paper, an analysis of the impact of noise on edge detection was conducted as well as a comparative analysis of the impact of noise on the effectiveness of the proposed threshold value estimation approach [9]. For the analysis of a medical images dataset together with its appropriate reference, an ideal-edge image (ground truth) was used. The analysis was performed for the noise types, namely salt and pepper, speckle, Rician, and Gaussian. For each noise type, a different intensity, i.e., different concentrations of noise in the image (0.01, 0.05, and 0.1, respectively), was used. The images were categorized as per their complexity (low, medium, and high), which was determined based upon the spatial information in the image. The results of the analysis show that the proposed approach was quite suitable when images affected by noise are concerned, particularly so when the Canny operator was applied. The approach has demonstrated remarkable resilience across the varied terrains of noise. Also, the findings not only underscore the importance of a tailored threshold value estimation method but also highlight its adaptability to different noise scenarios. This adaptability is particularly vital in the real-world application of edge detection, where images often contend with a multitude of noise sources. Furthermore, the categorization of images into complexity classes based on their spatial characteristics has enriched our understanding of noise’s impact. We observed that the effectiveness of the proposed approach remains consistent, regardless of an image’s complexity. This observation bodes well for practical applications, as real-world images are seldom uniform in their spatial characteristics.

The results provide a good analysis and a good comparison for further research efforts, such as the optimization of the access parameters with the help of machine learning for image filtering in the presence of noise. The findings presented in this study offer a strong foundation for future research endeavors, in particular, the application of machine learning techniques for the optimization of edge detection parameters in noisy conditions. Machine learning’s adaptive capabilities may offer a dynamic solution to the persistent challenge of noise in image processing. This avenue of research has the potential to refine the existing techniques achievable in real-world applications.

The direction of future research will be the additional optimization of algorithms over a dataset consisting of a larger number of images, as well as specialized images such as MRI, CT, satellite images, etc. and also the application of new optimization techniques, especially those based on deep learning and machine learning.

## Figures and Tables

**Table 1:** Characteristics of the edge detection model.

Characteristic	DexiNet	LDC	CATS	RS9	GS
Category	DL	DL	DL	Parameter optimization (indirect DL)	Parameter optimization (indirect DL)
Computational complexity	High	Medium	High	Variable: depends on the size of the search space and the number of hyperparameters	Medium: depends on the number of combinations in the search space
Time requirements’	High training requirements	Medium training requirements	High training requirements	Low training requirements	Medium training requirements
impact on model performance	Directly affects performance	Directly affects performance	Directly affects performance	Indirectly through optimization	Indirectly through optimization
Application	Specific tasks: Edge detection in images, computer vision	Specific tasks: Edge detection in images, computer vision	Specific tasks: Edge detection in images, computer vision	Adaptable to any ML model for hyperparameter optimization, e.g., edge detection	Adaptable to any ML model for hyperparameter optimization, e.g., edge detection

## Data Availability

The data supporting the findings of this study are derived from the Kaggle website which is publicly accessible at https://www.kaggle.com/datasets/beosup/lung-segment (accessed on 12 November 2024) for the lung segmentation dataset, https://www.kaggle.com/datasets/nikhilroxtomar/brain-tumor-segmentation (accessed on 12 November 2024) for the brain tumor segmentation dataset, and https://www.kaggle.com/datasets/andrewmvd/drive-digital-retinal-images-for-vessel-extraction (accessed on 12 November 2024) for the Retina dataset. The dataset includes images and their ground truth edge maps used in our analysis. Simulated noisy images and the results of the edge detection experiments can be obtained from the corresponding author upon reasonable request.

## References

[1] Gonzalez R.C., Woods R.E. (2008). Digital Image Processing.

[2] Lone A.H., Siddiqui A.N. (2018). Noise models in digital image processing. Glob. Sci. Tech.

[3] Thakur R.S., Chatterjee S., Yadav R.N., Gupta L. (2021). Image de-noising with machine learning: A review. IEEE Access.

[4] Oszust M., Bielecka M., Bielecki A., Stepień I., Obuchowicz R., Piórkowski A. (2022). Blind image quality assessment of magnetic resonance images with statistics of local intensity extrema. Inf. Sci..

[5] Bielecka M., Bielecki A., Obuchowicz R., Piórkowski A., Krzhizhanovskaya V.V. (2020). Universal measure for medical image quality evaluation based on gradient approach. Computational Science—ICCS 2020, Lecture Notes in Computer Science.

[6] Thakur R.S., Yadav R.N., Gupta L. (2019). State-of-art analysis of image denoising methods using convolutional neural networks. IET Image Process..

[7] Hajipour K., Mehrdad V. (2021). Edge detection of noisy digital images using optimization of threshold and self-organized map neural network. Multimed. Tools Appl..

[8] Fan L., Zhang F., Cao Z. (2019). Brief review of image denoising techniques. Vis. Comput. Ind. Biomed. Art.

[9] Buades A., Coll B., Morel J.M. (2005). A review of image denoising algorithms, with a new one. Multiscale Model. Simul..

[10] Marques O. (2011). Practical Image and Video Processing Using MATLAB.

[11] Maksimovic V., Petrovic M., Savic D. (2021). New approach of estimating edge detection threshold and application of adaptive detector depending on image complexity. Optik.

[12] Li H., Liao X., Li C. (2011). Edge detection of noisy images based on cellular neural networks. Commun. Nonlinear Sci. Numer. Simul..

[13] Bevandić P., Krešo I., Oršić M., Šegvić S. (2022). Dense open-set recognition based on training with noisy negative images. Image Vis. Comput..

[14] Thirumavalavan S., Jayaraman S. (2016). An improved teaching–learning based robust edge detection algorithm for noisy images. J. Adv. Res..

[15] Teng X., Zhang J., Li H., Liu Y., Mei J., Yang Q., Liu Z., Tang J., Zhou H. Adaptive edge detection of noisy images based on the fusion of grayscale and phase consistency. Proceedings of the SPIE 12065, AOPC 2021: Optical Sensing and Imaging Technology.

[16] Song J., Jiao W., Lankowicz K., Cai Z. (2022). A two-stage adaptive thresholding segmentation for noisy low-contrast images. Ecol. Inform..

[17] Maksimovic V., Milosevic M., Jaksic B., Petrovic M. Impact of brightness and complexities to edge detection with Roberts and Canny operator on compressed images. Proceedings of the International Scientific Conference—UNITECH 2020.

[18] Sekehravani E.A., Babulak E., Masoodi M. (2020). Implementing canny edge detection algorithm for noisy images. Bull. Electr. Eng. Inform..

[19] Ruslau M.F.V., Pratama R.A., Nurhayati, Asmal S. (2019). Edge detection in noisy images with different edge types. IOP Conf. Ser. Earth Environ. Sci..

[20] Baltierra S., Valdebenito J., Morales M.M. (2022). Edge detection in images with multiplicative noise using the Ant Colony System algorithm. Eng. Appl. Artif. Intell..

[21] Li W., Zhang L., Wu C., Zhenxiang C., Chao N. (2022). A new lightweight deep neural network for surface scratch detection. Int. J. Adv. Manuf. Technol..

[22] Obuchowicz R., Piórkowski A., Urbanik A., Strzelecki M. (2019). Influence of acquisition time on MR image quality estimated with nonparametric measures based on texture features. Biomed Res. Int..

[23] Maksimovic V., Jaksic B., Petrovic M., Palevic P. (2019). New approach to edge detection on different levels of wavelet decomposition. Comput. Inform..

[24] Bergstra J., Bengio Y. (2012). Random search for hyper-parameter optimization. J. Mach. Learn. Res..

[25] Kim S., Jung M., Park J. (2024). A study of tool wear measurement using image processing. J. Korea Robot. Soc..

[26] Hu G., Saeli C. (2024). Enhancing deep edge detection through normalized Hadamard-product fusion. J. Imaging.

[27] BenHajyoussef A., Saidani A. (2024). Recent advances on image edge detection. Digital Image Processing: Latest Advances and Applications.

[28] Sun R., Lei T., Chen Q., Wang Z., Du X., Zhao W., Nandi A.K. (2022). Survey of image edge detection. Front. Signal Process..

[29] Raj D.M.D., Shanmuganathan H., Geetha A., Keerthika V. EGF: An Improved Edge Detection Model for Low-Resolution Images. Proceedings of the 2nd International Conference on Futuristic Technologies (INCOFT).

[30] Yan J., Zhang L., Luo X., Peng H., Wang J. (2022). A novel edge detection method based on dynamic threshold neural P systems with orientation. Digit. Signal Process..

[31] Kalbasi M., Nikmehr H. (2020). Noise-robust, reconfigurable Canny edge detection and its hardware realization. IEEE Access.

[32] Soria X., Sappa A.D., Humanante P., Akbarinia A. (2023). Extreme inception network for edge detection. Pattern Recognit..

[33] Soria X., Pomboza-Junez G., Sappa A.D. (2022). LDC: Lightweight Dense CNN for edge detection. IEEE Access.

[34] Huan L., Xue N., Zheng X., He W., Gong J., Xia G.-S. (2021). Unmixing convolutional features for crisp edge detection. IEEE Trans. Pattern Anal. Mach. Intell..

[35] Tsai M.J., Lin P.Y., Lee M.E. (2023). Adversarial attacks on medical image classification. Cancers.

